# Personal

**Published:** 1911-02

**Authors:** 


### PERSONAL

---

DR. GLENTWORTH REEVE BUTLER, of Brooklyn, was chosen vice-president of the New York Academy of Medicine, at the annual election held in December, 1910.

---

DR. H. H. GLOSSER, of Buffalo, announces the removal of his office from 131 Allen Street, to 404 Franklin Street, where he will continue the practice of the late Dr. Julius Pohlman. Hours: 9 to 1 and by appointment.

